# A snapshot of Ischemic stroke risk factors, sub-types, and its epidemiology: Cohort study

**DOI:** 10.1016/j.amsu.2020.09.016

**Published:** 2020-09-16

**Authors:** Khaled Z. Alawneh, Majdi Al Qawasmeh, Liqaa A. Raffee, Bashar Abuzayed, Diab A. Bani Hani, Khalid Mohamed Abdalla, Asma'a Mohammad Al-Mnayyis, Jehad Fataftah

**Affiliations:** a Department of Diagnostic Radiology and Nuclear Medicine, Faculty of Medicine, Jordan University of Science and Technology, Irbid, Jordan; b Division of Neurology, Department of Neuroscience, Faculty of Medicine, Jordan University of Science and Technology, Irbid, Jordan; c Department of Accident and Emergency Medicine, Faculty of Medicine, Jordan University of Science and Technology, Irbid, Jordan; d Neurosurgeon, The Specialty Hospital, Amman, Jordan; e Department of Anesthesia and Recovery, Faculty of Medicine, Jordan University of Science and Technology, Irbid, Jordan; f Department of Diagnostic Radiology and Nuclear Medicine, Jordan University of Science and Technology, Jordan; g Radiology- Yarmouk University, Faculty of Medicine, Department of Clinical Sciences. (Shafiq Irshidat St, Irbid 21163, Jordan; h Hashemite University, Radiology Department, Jordan

**Keywords:** Diabetes mellitus, Epidemiology, Hypertension, Risk factors, Stroke, Sub-types

## Abstract

The burden of stroke can be substantially studied by establishing the functional consequences of stroke and its predictors on the population, economy and to guide rehabilitation efforts. This study aims to determine the subtypes, risk factors, and epidemiology of stroke in Jordan. **Methods**: A retrospective cohort study design was carried out to determine the risk factors and subtypes of stroke during 2017–2018. The study sample included 176 ischemic stroke patients of the King Abdullah University Hospital. Data was collected through medical records, which was then statistically analysed through frequencies and percentages. **Results:** Total 176 cases were identified out of which 101 (57.38%) were males and 75 (42.61%) were females and male to female ratio was 1.9:1. Hypertension was the commonest risk factor identified (50.56%), followed by diabetes mellitus (19.88%), hyperlipidaemia (15.34%), coronary artery disease (6.25%), atrial fibrillation (4.54%), and past history of stroke (1.13%). Risk factors such as hypertension (p = 0.007), diabetes (p = 0.000), coronary artery disease (p = 0.000) were significantly associated with subtypes of ischemic strokes. **Conclusion:** The study concludes that mean age of men was higher as compared to women in small vessel occlusion. The risk of ischemic stroke in patients with dyslipidaemia, diabetes mellitus, and hypertension was higher in middle-aged and old patients.

## Highlights

• A Snapshot of Ischemic Stroke Risk Factors, Sub-Types, and its Epidemiology.
• Risk of ischemic stroke is with dyslipidaemia, diabetes mellitus, and hypertension.
• Control and treatment of hypertension and dyslipidaemia should be prioritized.
• Risk of ischemic stroke is higher among middle and old aged patients.

## 1 Introduction

The rapidly developing changes in the epidemiology is an important global concern. A significant increase in the occurrence of stroke events has been identified in recent studies [1]. According to the World Health Organization, the incidences of stroke have been doubled in the last four centuries specifically in low and middle income populations of developing countries [2]. Globally, the stroke burden is escalating and it is anticipated that stroke will be ranked 4th in the disease burden by 2020 [[3], [4], [5]]. Ischemic stroke is classified as one of the sub-types of stroke. The interruption of the blood supply to a part of the brain causes ischemic stroke, which results in sudden loss of function [6]. Approximately 80% stroke cases are caused by ischemic strokes [[7], [8], [9], [10], [11]]. Intracerebral haemorrhage and subarachnoid haemorrhage are the sub-types of haemorrhage stroke [8]. A wide range of stroke risk factors have been recognized, which are essential for the development of strategies to prevent primary and secondary stroke. Modifiable and non-modifiable risk factors are included in the common causes of stroke [12]. Modifiable risk factors include high blood cholesterol, sedentary lifestyle, smoking, alcohol consumption, atrial fibrillation, cardiovascular diseases, and diabetes mellitus [[13], [14], [15]]. However, there are relatively few non-modifiable risk factors, which usually include age and gender.

Ischemic stroke is proved to be the most prevalent type of stroke; however, in Caucasian populations, the number of patients of haemorrhagic stroke is higher. Intravenous thrombolysis was used for treating the stroke in Thailand. The launch of stroke fast track system has assured the speedy stroke treatment throughout the country [16]. Low density lipoprotein (LDL) cholesterol which causes ischemic stroke is negatively related to Statin therapy but more verification is required to know whether or not LDL cholesterol causes ischemic stroke subtypes. Additionally, the risk factors of ischemic stroke and its subtypes in terms of high-density lipoprotein cholesterol and triglycerides is still not known [17]. Many loci displayed different relation and patterns of pleiotropy for stroke subtypes [18].

In European populations, Ischemic stroke is caused by cardio embolism, while it usually occurs in large artery atherosclerosis in Asian populations [19]. Undetermined subtype includes largest number of ischemic strokes followed by small artery disease (SAD), cardio embolism (CE), and large artery disease (LAD). After doing adjustment in age, the high level occurrence of subtypes SAD and LAD were reported in Middle East countries. The larger portion of undetermined stroke cases in this study is because of the less access to diagnostic studies [20]. This pinpoints that stroke needs to be measured by exact and well-mannered methods with respect to Middle Eastern population. Public awareness should be increased with respect to stroke and its risk factors should be provided to assist the population to comprehend the detrimental effects of stroke [21]. Countries which share same demographic or socioeconomic conditions need to learn from each other how to minimize stroke occurrence and case-mortality rates [22]. Therefore, this study aims to determine the subtypes, risk factors, and epidemiology of ischemic stroke in Jordan. Following are the objectives of this study:

• To determine the subtypes of ischemic stroke in the study population.
• To determine the most prevalent risk factors among ischemic stroke patients.
• To describe the prognosis of patients with ischemic stroke.

The current study is significant as it determines the most prevalent risk factors and subtypes of stroke which will help authorities to plan health services and resources distribution, and to devise prevention strategies. The burden of this disease can be substantially studied by establishing the functional consequences of stroke and its predictors on the population and economy to guide rehabilitation efforts. Identification of post-stroke depression as a regular complication assists in planning strategies for diagnosing and initial treatment of this condition, which will positively impact the outcome and recovery of stroke patients.

## 2 Methods

### 2.1 Registration and ethics

The study is conducted in accordance with the declaration of Helsinki under the Unique Identifying Number (UIN).

### Ethical approval

The study was approved by the Ethics Committee of King Abdullah University Hospital.

### 2.2 Protocol

The study published its protocol prior which is based on one of the previously published studies [23].

### 2.3 Patient involvement in research

The patient involvement in this study was based on accountability of people with the risk factors associated with stroke that is taken as the greatest relevance. All information related to each subject was de-identified as each patient was assigned a particular code, which was used for patient's identification during the study. All records of patients were locked and password protected with KA file number. The de-identified database was used to perform all the analysis.

### 2.4 Study design

A retrospective cohort study design was carried out to determine the risk factors and subtypes of stroke.

### 2.5 Setting

The sample population of this study included ischemic stroke patients admitted in King Abdullah University Hospital during the period 2017–2018.

### 2.6 Participants

All the patients with the ICD-10 diagnosis of ischemic stroke, admitted in King Abdullah University Hospital during the period 2017–2018 included study population. Total 176 ischemic stroke cases were identified and were found eligible to be included in this study on the basis of the following inclusion criteria: middle and old aged patients between 30 and 75 years, diagnosed with the below mentioned risk factors and subtypes of ischemic stroke were included in the study. The features of patients were identified on the basis of detailed review of patients’ diagnostic details and relevant reports. However, patients with insufficient medical record for diagnosis, those diagnosed with haemorrhagic stroke, and patients with the final diagnosis of TIA were excluded from this study.

### 2.7 Recruitement

The medical records of patients including their age, gender and ischemic stroke risk factors including the hypertension, diabetes mellitus, hyperlipidaemia, coronary artery disease, atrial fibrillation, smoking status, and valvular heart diseases of ischemic subtypes were obtained.

In addition, the subtypes of ischemic stroke were determined through the TOAST criteria, which involves five major categories of TOAST classification: large-artery atherosclerosis (LAA), cardio-embolism, small artery occlusion, stroke of other determined cause and that of undetermined cause. However, any one of the below illustrated information was required for strokes undetermined causes:

a) The reports failed to provide the cause despite of a detailed evaluation.
b) The reports failed to determine a most likely cause due to the identification of 1> possible cause.

A statistical program (SPSS, IBM, Chicago) version 23.0 was used to analyse the collected data. Continuous data were presented in the form of mean and standard deviation; whereas, categorical variables were displayed in frequencies and percentages.

## 3 Results

Total 176 patients were identified: 101 (57.38%) were males and 75 (42.61%) were females and the male to female ratio was 1.9:1. Total of 131 patients (74.4%) belong to the old age group, in comparison to those of the middle age (25.5%). Table 1 presents frequencies and percentages of different risk factors for all ischemic strokes. Hypertension was the commonest risk factor identified among the cases (50.56%), followed by diabetes mellitus (19.88%), hyperlipidaemia (15.34%), coronary artery disease (6.25%), atrial fibrillation (4.54%), and past history of stroke (1.13%). Although some of the patients had two risk prognostic factors (Brain aneurysms or arteriovenous malformations, infections, anxiety, high stress level, depression), but the overlapping comorbid conditions were ignored. Moreover, only important risk factors that need to be prevented have been considered in this study. Table 2 presents mean age of male and female patients in different subtypes of stroke. The findings reported that highest mean age for both male (64.6) and female (70.9) patients was found in small vessel occlusion.

**Table 1:** Participant details for risk factors associated to ischemic strokes.

Variables	Frequency (%)
**Gender**	
**Male**	101 (57.38%)
**Female**	75 (42.61%)
**Age**	
**Middle aged patients (36**–**55 years)**	45 (25.5%)
**Old aged patients (55 years and above)**	131 (74.4%)
**Risk factors**	
**Hypertension**	89 (50.56%)
**Diabetes mellitus**	35 (19.88%)
**Hyperlipidaemia**	27 (15.34%)
**Coronary artery disease**	11 (6.25%)
**Atrial fibrillation**	8 (4.54%)
**Past history of stroke**	2 (1.13%)
**Smoking**	1 (0.005%)
**Valvular heart disease**	3 (1.7%)

**Table 2:** Mean age for males and females in Different Subtype of Stroke.

Ischemic Stroke Subtypes	Mean age (Male)	*P*-value	Mean age (Female)	*P*-value
**Determined Causes**				
**Large artery atherosclerosis**	61.6	0.021	68.2	0.011
**Small vessel occlusion**	64.6		70.9	
**Cardio embolic**	59		65.1	
**Other determined causes**	52		69	
**Undetermined Causes**	56.2		48.1	

**Fig. 1:**
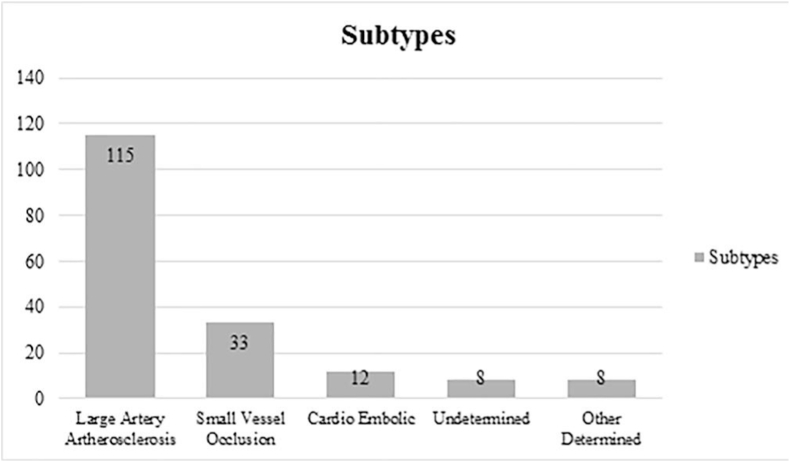
Etiologic subtypes of ischemic stroke.

Fig. 1 and Table 3 reveal the etiologic subtypes of ischemic strokes and their distribution based on risk factors among patients respectively. Total 115 patients were reported with large artery atherosclerosis, followed by small vessel occlusion (33), cardio embolic (12), undetermined (8), and other determined (8) (Fig. 1). Risk factors including hypertension (p = 0.007), diabetes (p = 0.000), coronary artery disease (p = 0.000) were significantly associated with subtypes of ischemic strokes (Table 3). However, other risk factors including hyperlipidaemia (p = 0.500), BMI (p = 0.89), and atrial fibrillation (p = 0.49) were found insignificantly associated with subtypes of ischemic strokes.

**Table 3:** Distribution of risk factors among major categories of ischemic stroke.

Risk factors	Large artery atherosclerosis	Small Vessel Occlusion	Cardio Embolic	Undetermined	*P*-value
**Hypertension**	31 (35%)	20 (22.9%)	13 (14.9%)	23 (26.4%)	0.007
**Diabetes mellitus**	14 (40%)	8 (22%)	4.9 (14%)	8 (23.4%)	0.000
**Hyperlipidaemia**	10 (36%)	5 (19%)	4 (14%)	8 (28%)	0.500
**Coronary artery disease**	2 (20%)	5 (48%)	1 (12%)	3 (18%)	0.000
**BMI**	8 (28%)	10 (36%)	4 (18%)	1 (18%)	0.89
**Atrial fibrillation**	2 (24%)	4 (39%)	1 (18%)	1 (18%)	0.49

## 4 Discussion

The study determined the subtypes, risk factors, and epidemiology of stroke in Jordanian patients. Hypertension was the commonest risk factor identified in most cases (50.56%), followed by diabetes mellitus (19.88%), hyperlipidaemia (15.34%), coronary artery disease (6.25%), atrial fibrillation (4.54%), and past history of stroke (1.13%). It was observed that atrial fibrillation results in static blood flow and contractile dysfunction leading to brain embolism and thrombus formation. Additionally, due to structural remodelling of the atrium, the risk of thromboembolism inclines as atrial cardiopathy decreases. The study findings further suggested that the risk factors such as hypertension, DM, and hyperlipidaemia are high in patients with large artery atherosclerosis. These findings are in line with the findings proposed by Renjen, Beg and Ahmad [24], according to which the prevalence of the above listed risk factors was high among those suffering from large-artery atherosclerosis. In contrast to this, Bahou, Ajour and Jaber [25] who showed that the prevalence of the above risk factors was high among patients with lacunes.

Another important finding of this study was the positive and significant relationship between hypertension and occurrence of ischemic stroke. Diabetes mellitus was the second most dominant factor to be associated with incidence of stroke. This finding has been corroborated by previous studies who found a positive significant association between diabetes mellitus and incidence of stroke [[26], [27], [28]]. Hosaini et al. [26] shows 29.3% of all stroke patients having hyperglycemia. The prevalence of diabetes in ischemic stroke patients was 78.1% with large artery atherosclerosis, followed by small vessel occlusion (62.4%), and cardio embolic (60%). These findings were supported by Aquil et al. [29] who found 75% ischemic heart disease and 50% atrial fibrillation with respect to cardio embolic subtype.

Findings of the current study further indicate that most of the patients suffering from the ischemic heart diseases aged above 55 years. These findings are in line with those provided in the study of Sun [30] according to which the incidences of stroke are frequent after the age of 55 years. Yousuf and Young [31] further supported the idea and stated the prevalence of ischemic stroke risk factors increases by the age of 65.

Both primary and secondary stroke can be prevented by controlling diabetes and might reduce the mortality too. Improvements in dietary pattern and nutrition in diabetic patients can reduce the occurrence of cardiovascular disease substantially beside weight management [29]. Therefore, nutritional patterns and dietary aspects must be integrated into any national prevention strategy.

The results indicate that highest mean age was found in cases of small vessel occlusion for both male and female patients. However, previously no association was found in male and female patients between diabetes mellitus history and stroke outcome. Women had lower mortality, dependency rates, and recurrence, while this study shows a higher prevalence of diabetic mellitus in women as compared to men. The control and management of diabetic mellitus levels in patients can enhance positive consequences for female stroke patients, and this might have contributed to the lower risk of negative consequences in female patients. Lastly, 115 patients were reported with large artery atherosclerosis, followed by small vessel occlusion, cardio embolic, undetermined, and other determined. All risk factors were significantly associated with subtypes of ischemic strokes at 5% level of significance.

Due to incompleteness of some parameters, including height, weight exercise, and occupation, the current study was limited by the quality of the documentation of medical. Diagnoses were not documented appropriately by the physician. To overcome this limitation, all reports should be considered when attending physician specifically for medical records reporting diagnosis of unidentified stroke type. These risk factors must be taken into account for decision-making when establishing preventing strategies. Finally, the results of this study may not be generalized to the entire population due to the small sample size.

The study has determined the subtypes, risk factors, and epidemiology of stroke in Jordanian patients and found hypertension as the commonest risk factor among the subtypes. The study concludes that mean age was higher among men as compared to women in small vessel occlusion. Among the etiologic subtypes of ischemic strokes and distribution on risk factors, large artery atherosclerosis was the commonest etiologic subtypes reported in 115 patients. Risk factors including hypertension, diabetes, and coronary artery disease are positively and significantly associated with ischemic strokes subtypes (p < 0.05).

The risk of ischemic stroke in patients with dyslipidaemia, diabetes mellitus, and hypertension was higher as compared to haemorrhagic stroke and this situation calls for a prevention plan at the national level which should focus mainly on lifestyle factors such as body mass index, smoking, diet, and exercise. Priority should be given to the control and treatment of hypertension and dyslipidaemia using medical management and lifestyle modifications in the studied patients. Therefore, there is a need for adoption of a population-based or cohort study of stroke in the region. There is a definite need for the development of population or hospital-based stroke registry at national or regional levels.

## Provenance and peer review

Not commissioned, externally peer reviewed.

## Funding

This research received no external funding.

## Declaration of competing interest

The authors declare no conflict of interest.
